# Performance of emergency medical technician course students regarding adherence to the rules of guidance and driving on crossing roads outside the city in Urmia 

**Published:** 2019-08

**Authors:** Nader Aghakhani, Gholamreza Esmhoseini, Narges Rahbar, Abbas Zarei, Yedgar Fattahi

**Affiliations:** ^*a*^Patient Safety Research Center, Urmia University of Medical Sciences, Urmia, Iran.; ^*b*^Forensic Medicine organization, Urmia, Iran.; ^*c*^Patient Safety Research Center, Urmia University of Medical Sciences, Urmia, Iran.

## Abstract

**Background:**

In terms of accidents and traffic accidents, Iran is one of the countries with the highest incidence and mortality rate. According to estimates, many people in Iran die from road traffic accidents and this proportion is also high compared to the world and the region estimations. Considering the importance of the role of human factors in the occurrence of traffic accidents, the best way is preventive and educational programs, as is expected from profession rescuers to be in compliance with road safety regulations since entering the profession due to their insight into the dangers. Given that medical emergency students will be future roadside assistants; the question arises as to whether they are self-regulating agents?

**Methods:**

The present study was conducted on 46 students’ performance. Since the samples were homogeneous, their demographic characteristics were virtually identical and not considered in the research.

**Results:**

The results of this study showed that the performance of the research samples in respect to driving guidance is relatively good. Paying attention while passing the street with 56.5% had the highest level of performance and non-observance of food when crossing the street and not moving alongside the streets with 17.4% were the least cases.

**Conclusions:**

Considering the importance of the role of human factors in the occurrence of accidents and driving accidents and the high contribution of traffic accidents to mortality and morbidity in our society, the best and most cost-effective way to reduce the occurrence of such incidents is to apply educational strategies and preventive programs. It is essential to observe the rules and ethical beliefs of the authorities and the rescuers. However, in order to obtain better results, increasing the number of samples and observing their performance seems more appropriate.

**Keywords:**

Emergency medical technician, Students, Adherence, Guidance, Urmia

